# CRISPR/Cas9–mediated MMP-9 silencing inhibits bladder cancer T24 cell invasion and migration in vitro

**DOI:** 10.1016/j.clinsp.2025.100842

**Published:** 2025-11-22

**Authors:** Fabio Pescarmona Gallucci, Juliana Alves de Camargo, Nayara Izabel Viana, Ruan Cesar Aparecido Pimenta, Vanessa Ribeiro Guimarães, Patrícia Candido, Katia Ramos Moreira Leite, William Carlos Nahas, Sabrina Thalita dos Reis

**Affiliations:** a Laboratório de Investigação Médica 55 (LIM55), Hospital das Clínicas, Faculdade de Medicina, Universidade de São Paulo (HCFMUSP), São Paulo, SP, Brazil; b Uro-Oncology Group, Urology Department, Instituto do Câncer do Estado de São Paulo (ICESP), São Paulo, SP, Brazil; c Universidade do Estado de Minas Gerais (UEMG), Passos, MG, Brazil; d Precision Immunology Institute, Department of Immunology and Immunotherapy, and Tisch Cancer Institute, Icahn School of Medicine at Mount Sinai, NY, USA; e Instituto Moriah de Ciência e Educação (MISE), Hospital Moriah, São Paulo, SP, Brazil

**Keywords:** Bladder cancer, Matrix metalloproteinases, CRISPR/Cas9, MMP-9, Extracellular Matrix, Invasion, Migration

## Abstract

**Purpose:**

Bladder Cancer (BCa) ranks as the tenth most common cancer worldwide, with high morbidity and mortality. Matrix Metalloproteinases (MMPs), especially MMP-9, are associated with tumor progression and metastasis. This study aimed to evaluate the effects of MMP-9 silencing using CRISPR-Cas9 in T24-luc bladder carcinoma cells.

**Methods:**

Guide RNAs (sgRNA1 and sgRNA2) targeting MMP-9 were cloned into pX-330 plasmids and transfected into T24-luc cells. Gene and protein expression were analyzed via RT-qPCR and Western blotting. Functional assays included flow cytometry for proliferation and apoptosis, colony formation, wound healing, and Matrigel™ invasion assays.

**Results:**

sgRNA2 significantly reduced MMP-9 gene expression, while both sgRNAs reduced protein expression. Edited cells showed decreased proliferation and colony formation, increased apoptosis, and reduced migration and invasion capacity.

**Conclusion:**

CRISPR-Cas9-mediated silencing of MMP-9 inhibited cell proliferation, migration, invasion, and increased apoptosis in BCa cells, supporting that MMP-9 has an important effect on the progression of bladder cancer.

## Highlights

• CRISPR/Cas9 effectively silenced MMP-9 in bladder cancer cells.
• MMP-9 silencing reduced T24 cell proliferation and colony formation.
• Edited cells showed increased apoptosis after MMP-9 knockdown.
• Migration and invasion capacity were strongly inhibited by MMP-9 silencing.
• MMP-9 is a potential therapeutic target in bladder cancer progression.

## Introduction

Bladder Cancer (BCa) is the 10th most commonly diagnosed cancer and the second most frequent genito-urinary cancer worldwide, with an estimated 573,000 new cases and 213,000 deaths in 2020 globally,^1^ while 82,290 new cases and 16,710 deaths were estimated in 2023 in the United States.^2^ It is four times more frequent among men than women and is correlated with tobacco smoking and occupational exposures (painting, rubber, and aluminum industries).^1^ Most of these cancers are urothelial carcinomas (over 90 %), with higher incidences observed in the elderly population.^3^ In addition, most (75 %) BCa are Non-Muscle Invasive (NMIBC) at first diagnosis (pTa, pT1, or Carcinoma In Situ [CIS]), while 25 % have Muscle-Invasive Disease (MIBC) or metastatic disease.^4^^,^^5^ High recurrence and progression rates are observed in NMIBCs, with recurrence rates ranging from 15 %‒61 % at one-year and 31 %‒78 % at five-year follow-ups, and up to 45 % (depending on risk stratification) of NMIBCs will progress into MIBCs at five years.^6^ Treated MIBC patients present poorer long-term survival rates (29 %‒69 %) than NMIBC patients.^7^, ^8^, ^9^ Recurrence and progression from NMIBC to MIBC, which require more invasive, complex, and costly treatments, negatively impact the quality of life^10^^,^^11^ and increase healthcare costs per patient.^11^ Among cancers in the United States, BCa has the highest per-patient treatment costs and the fifth-highest overall cost.^12^^,^^13^ Therefore, it is essential to characterize the molecular mechanisms of BCa tumorigenesis, progression, and recurrence that lead to the development of new therapeutic options to achieve long-term cost and quality-of-life benefits through reduced tumor recurrence and progression rates.

Epithelial-to-Mesenchymal Transition (EMT) is a primary process involved in tumor progression and metastasis. Matrix Metalloproteinases (MMPs) play a central role in this process due to their ability to degrade the Extracellular Matrix (ECM) and basement membranes.^14^^,^^15^ In addition, the expression of MMPs has been correlated with cancer progression, metastasis, and relapse in multiple types of solid tumors, including colorectal, breast, prostate, and bladder cancer.^16^, ^17^, ^18^, ^19^ Among the MMPs, the MMP-9 degrades type-IV collagen, a significant basement membrane component. Therefore, MMP-9 has been the subject of several studies regarding the role of MMPs in bladder cancer as a screening/diagnostic and prognostic biomarker, making it an attractive therapeutic target in BCa.^20^^,^^21^

In recent years, the CRISPR/Cas9 technique has emerged as a flexible, efficient, specific, and simple genome editing tool for both research and disease treatment. The system is composed of two parts: a “single guide RNA” (sgRNA), which recognizes a specific target sequence in the genome, and the Cas9 protein, which promotes de double-strand break of the target DNA.^22^

In the present study, the authors investigated the effect of blocking the MMP-9 expression by genome editing using the CRISPR/Cas9 technique on the migration and invasion capabilities of human T24-Luc bladder cancer cells.

## Materials and methods

### Cell culture

The human bladder carcinoma cell line T24, previously transfected with a viral vector containing the firefly luciferase gene (T24-Luc), was used in the experiment. The cells were cultured in Minimum Essential Media (MEM) containing 10 % fetal bovine serum and 1 % antibiotic and antimycotic solution (Sigma Co, USA). They were maintained in a humidified incubator at 37 °C and a 5 % CO_2_ atmosphere. This study was conducted in accordance with the ARRIVE guidelines, adapted for in vitro cell experiments.

### Plasmid construction

CRISPR/Cas9 plasmid was constructed using the px330 plasmid obtained through Addgene (www.addgene.org). This plasmid encodes SpCas9, GFP, ampicillin resistance and puromycin resistance sequences, and *BbsI* restriction site. Next, the sgRNA sequence targeting the MMP9 was synthesized using the technique described by Ran et al. These sequences have a high score, indicating a low probability of off-targets, as described by the author who developed the methodology that the authors use.^23^ The sgRNAs are from EXON 1 of MMP-9. Therefore, the final plasmid was constructed by inserting annealed oligo pairs 5′-caccGGAAGGGTGGACTGGCGCTGTC-3′ and 5′-aaacGACAGCGCCAGTCCACCCTTCC-3′, ligated into the *BbsI* restriction site. Hereafter, a bacterial transformation of the px330-MMP9 plasmid was performed into Dh5α *E.coli*, with subsequent bacterial culture expansion in an ampicillin-containing medium, plasmid extraction, and validation through sequencing.

### Transfection in T24 cells

Polymer-mediated transfection was conducted by seeding 2 × 10^5^ T24-Luc cells in a 6-well plate the day before transfection. A mixture containing pDNA (px330-MMP9 plasmid) and the Xfect™ transfection reagent (Takara Bio Inc., Shiga, Japan) was prepared following the manufacturer’s protocol. First, transfection was performed by adding 7.5 µg plasmid to 100 µL of Xfect™ reaction buffer, followed by adding 2.25 µL of Xfect™ polymer. Next, the solution was added to the 6-well plate cultures in the incubator at 37 °C for 4 h. After that, the culture medium was replaced. Transfection success was validated through GFP microscopy.

### Proliferation and apoptosis assays by flow cytometry

In order to quantify the cellular proliferation and apoptosis after cell transfection, the Muse Cell Analyzer (Merck Millipore, Burlington, MA, USA) was used. After the transfection experiments, the cells were labeled with the Muse Ki67 Proliferation kit (MCH100114) and the Muse Annexin V & Cell Death kit (MCH100105), according to the manufacturer´s instructions.

### Colony formation assay

The T24-Luc cells were cultured in 6 cm Petri dishes (300 cells/dish) and incubated at 37 °C and 5 % CO^2^ atmosphere for ten days, distributed in three groups (control, sgRNA1, and sgRNA2). Following incubation, the culture medium was removed, and the cells were washed with PBS and fixed with a 3:1 methanol/acetic acid solution for 5 min. Next, the cells were stained with a 5 % crystal violet solution. The observed colony area (µm^2^) was measured using ImageJ® software (https://imagej.nih.gov/ij).

### RNA isolation and cDNA synthesis

According to the manufacturer’s protocol, RNA was extracted from cell cultures using the mirVana™ (Ambion, Austin, TX, USA) kit. Then, the purified samples were quantified using a spectrophotometer (Nanodrop DN-1000™, Thermo Scientific, Wilmington, DE, USA) and finally stored at −20 °C for future use.

Total RNA was diluted in nuclease-free water, constituting a total volume of 20 µL (50 ng/µL). Each reaction contained 4 µL of the RNA solution and 16 µL of the cDNA mix. RT reactions were performed using MultiScribe Reverse Transcriptase™ (Applied Biosystems, Foster City, CA, USA) enzyme. The reaction conditions were 25 °C for 10 min, 37 °C for 120 min, and 85 °C for 5 min. After the cycles, cDNA was stored at −20 °C until further use.

### Quantitative real-time PCR (RT-qPCR) and gene expression

Expression levels of the MMP9 gene were analyzed by RT-qPCR using an ABI 7500 Fast Real-Time PCR System™ (Applied Biosystems, Foster City, CA, USA). The target sequence was amplified in a 10 µL reaction containing 5 µL of TaqMan Universal PCR Master Mix™ (Applied Biosystems, Foster City, CA, USA), 0.5 µL of the specific primer, 2.5 µL of nuclease-free water, and 2 µL of cDNA. The PCR reaction conditions were 2 min at 50 °C, 10 min at 95 °C, forty 15-second cycles at 95 °C, and 1 min at 60 °C. In addition, a Human B2M (Beta-2-Microglobulin) assay (Applied Biosystems, Foster City, CA, USA) was used as an endogenous control. The experiments were performed in triplicate.

### Western blotting

Control and transfected cells were harvested and lysed using the RIPA Lysis Buffer™ (Merck KGaA, Darmstadt, Germany) and then centrifuged at 11,000 rpm for 20 min at 4 °C. Next, protein concentration levels were measured by spectrophotometry (Nanodrop DN-1000™, Thermo Scientific, Wilmington, DE, USA) using the Pierce™ 660 nm Protein Assay Reagent (Thermo Scientific, Wilmington, DE, USA), and the Laemmli buffer was added in a 1:2 proportion. The samples (50 µg/mL concentration) were run on a 10 % polyacrylamide gel at 80 V tension for 30 min and then 100 V for 90 min. After the gel run, the samples were transferred to a nitrocellulose membrane submerged in a buffer solution under refrigeration and 120 V tension for 90 min. Next, the membranes were blocked in 1 % BSA in TBS-T for 15 s. Next, antibody incubation was performed using the SNAP i.d.® Protein Detection System (Merck KGaA, Darmstadt, Germany) following the manufacturer’s protocol. Washed three times with TBS-T for 10 min, the blots were incubated with the secondary antibody following the same protocol. The results were visualized using the Luminata™ Western HRP Chemiluminescence Substrates (Merck KGaA, Darmstadt, Germany). The Alliance 4.7 (Uvitec, Cambridge) detection device was used for image acquisition.

### Wound healing cell scratch migration assay

10^5^ T24-Luc cells were cultured in 12-well plates containing non-transfected and transfected cells. The experiment was performed in triplicate. After 24 h, a straight linear wound was made using a sterile 200 µL micropipette tip to scratch the cell layer in each well. The distance between the two sides of the wound was measured and photographed at 0 h, 24 h, and 48 h. For each image, wound distance was measured using NIS Elements D 3.1 Software (Nikon). The migration distance was measured as a percentage of the initial distance at 0 h, based on measuring the area of risk using the formula: (A Initial – A Final)/(A Initial) × 100 = % wound closure/migration, where A is the measurement of the area between the edges of the scratch.^24^

### Matrigel™ invasion assay

The Matrigel™ invasion assay was performed in a chamber system consisting of polycarbonate membrane inserts with 8 µm pores (BioCoat Matrigel Invasion Chamber ‒ BD Biosciences, USA) placed in 24-well insert companion plates. The inserts were coated with a thin layer of 0.5 mg/mL Matrigel Basement Membrane Matrix (BD Biosciences, USA). The control (non-transfected) and the transfected T24-Luc cells (1 × 10^5^ in 750 µL of growth medium without FBS) were placed in the upper chamber, and 1 ml of growth medium containing 10 % FBS was placed in the lower chamber. The cells were incubated at 37 °C and allowed to invade through the Matrigel™ layer for 24 h. After incubation, the insert membranes were fixed with 75 % methanol. Next, the cells on the upper surface were removed with cotton-tipped swabs, and the invading cells on the lower surface were stained with 0.5 % crystal violet containing 20 % methanol. Finally, the stained cells were counted under an inverted microscope (10 fields per membrane). Each experiment was performed in triplicate.

### Statistical analysis

The statistical analyses were performed using GraphPad Prism 9.0 (GraphPad Software, CA, USA). Data was presented by mean ± SD (Standard Deviation) for quantitative variables. The Shapiro-Wilk test was used to assess the normality of the data. Student´s *t*-test and ANOVA test with Bonferroni correction (three or more groups) were used for group comparisons, except for the colony formation assay, where the Kruskal-Wallis test was used. The level of statistical significance was set to 5 % (*p* ≤ 0.05).

## Results

### MMP-9 knockout effect on cell proliferation and apoptosis

**Fig. 1:**
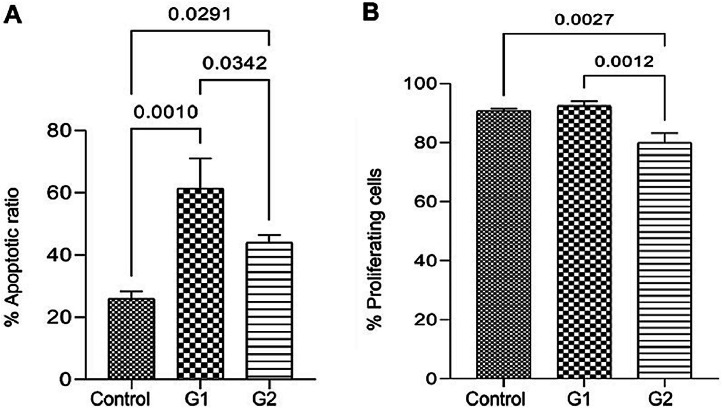
Effect of MMP-9 downregulation by CRISPR/Cas9 in flow cytometry assays with T24-Luc cells. (A) Percentage of apoptotic cells in control, sgRNA1 (G1), and sgRNA2 (G2) groups. (B) Percentage of proliferating cells in control, sgRNA1 (G1), and sgRNA2 (G2) groups. The p-values obtained from the statistical analysis are displayed above the bars in each panel. The error bar corresponds to the standard deviation of the sample (two-column fitting).

**Fig. 2:**
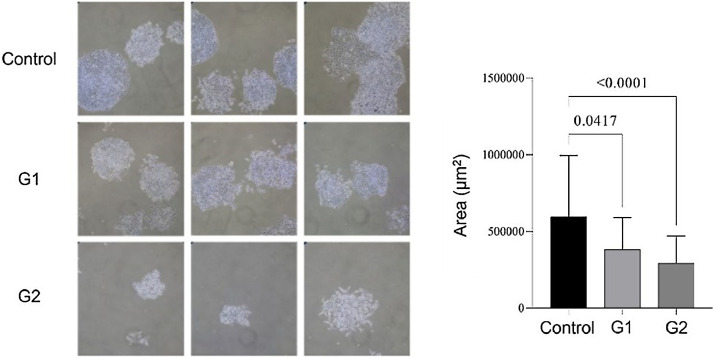
Colony formation assay. Compared to the control, lower colony formation capacity is observed in transfected T24-Luc cells. The p-values obtained from the statistical analysis are displayed above the bars in each panel. The error bar corresponds to the standard deviation of the sample (two column fitting).

Apoptosis analysis by flow cytometry showed that MMP-9 suppression after T24-Luc cell transfection caused a significant increase in the apoptosis rate for both sgRNAs (Control: 26.02 ± 2.31 vs. G1: 61.43 ± 9.68/G2: 44.05 ± 2.41; *p* = 0.001/ *p* = 0.029) (Fig. 1A). Meanwhile, the downregulation of MMP-9 significantly inhibited cell proliferation only when sgRNA2 was used, as shown in the proliferation assay (Control: 90.69 ± 0.83 vs. G1: 92.44 ± 1.61/G2: 80.02 ± 3.24; *p* > 0.05/ *p* = 0.002) (Fig. 1B). The colony formation capability of T24 cells was reduced after transfection for both sgRNAs (Control: 595,581 ± 400,139 vs. G1: 381,569 ± 206,747 vs. G2: 292,617 ± 177,554; *p* < 0.05) (Fig. 2).

### Inhibition of MMP-9 expression by CRISPR/Cas9

**Fig. 3:**
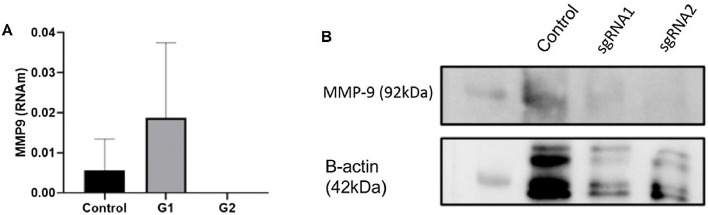
MMP-9 expression inhibition by CRISPR/Cas9. (A) After 24 h transfection, the cells were collected, and the level of MMP-9 expression was measured by RT-qPCR. No MMP-9 expression was detected using sgRNA2. (B) Western blotting for MMP-9 protein expression in T24 cells transfected with sgRNA 1 and sgRNA 2 plasmids. Low MMP-9 protein expression is observed for both sgRNAs (two-column fitting).

A total of two sgRNAs (sgRNA1 and sgRNA2) targeting MMP-9 were designed and inserted into the px-330 plasmid as previously described. Their effects on MMP-9 expression were determined by RT-qPCR after transfection. While sgRNA1 exhibited an increase in MMP-9 RNAm expression, sgRNA2 showed a knockdown effect on MMP-9 expression levels in T24-Luc cells compared to non-transfected control cells (Fig. 3A). In addition, the authors verified the protein expression levels of MMP-9 using western blotting. The results showed a significant reduction in protein expression for both sgRNAs (Fig. 3B).

### Inhibition of cell migration and invasion

**Fig. 4:**
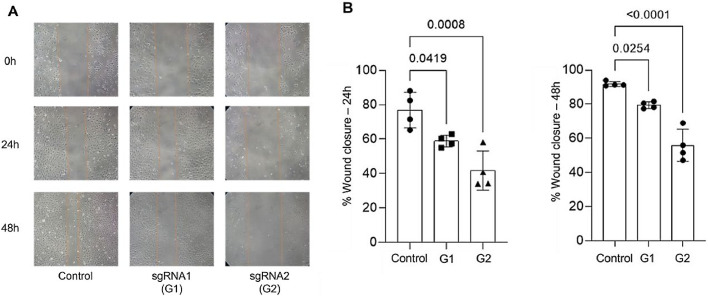
Wound healing assay. (A) Wound healing assay images were obtained by optical microscopy with a 10 × objective for the 24 h and 48 h times for both sgRNA1 and sgRNA2 and Control groups. (B) Decreased cell migration of T24 cells transfected with sgRNA1 (G1) and sgRNA2 (G2) compared to Control after 24 h and 48 h. The p-values obtained from the statistical analysis are displayed above the bars in each panel. The error bar corresponds to the standard deviation of the sample (two-column fitting).

**Fig. 5:**
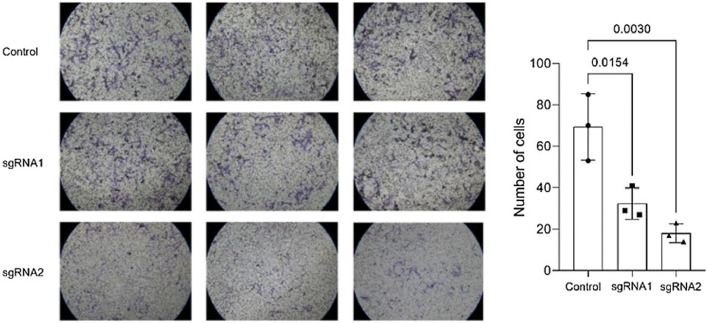
Matrigel™ invasion assay. A reduced average rate of cells invading the membrane post-transfection with sgRNA1 and sgRNA2, visualized at 10 × objective, is observed compared to the control. The p-values obtained from the statistical analyses are displayed above the bars in each panel. The error bar corresponds to the standard deviation of the samples (two-column fitting).

The authors conducted migration and invasion assays to evaluate the effect of MMP-9 knockdown on T24-Luc cell functions. Downregulation of MMP-9 expression successfully inhibited cell mobility, as shown by significant wound closure retardation (Table 1) (Fig. 4). In addition, T24-Luc invasion capability was also significantly impaired after transfection (G1: 32.33 ± 7.57 vs. Control: 69.33 ± 16.01; *p* = 0.015) e (G2: 18.0 ± 4.58 vs. Control: 69.33 ± 16.01; *p* = 0.003), as shown in the MatrigelTM Invasion Assay (Fig. 5).

**Table 1:** % Wound closure area at 24 h and 48 h.

	% Wound closure area			
	Control	G1 (sgRNA1)	G2 (sgRNA2)	p (Ctrl vs. G1/G2)
**24h**	76.90 ± 10.3	58.97 ± 3.41	41.79 ± 11.38	0.041 / <0.001
**48h**	91.81 ± 1.44	79.47 ± 1.99	55.91 ± 9.43	0.025 / <0.001

## Discussion

The present study examined the effects of reducing MMP-9 expression in T24-Luc bladder cancer cells using the CRISPR-Cas9 genome editing technique in an in vitro experimental setting. To investigate the functions of MMP-9 in T24 cells, the authors designed two sgRNAs targeting exon 1 of the MMP-9 gene. Transfection of T24-Luc cells with CRISPR-Cas9-sgRNA plasmids resulted in targeted genome-specific DNA cleavage, suppressing MMP-9 expression in vitro, decreasing cell survival and proliferation, and inhibiting cell migration and invasion, as verified by wound healing cell scratch migration assays and Matrigel™ invasion assays. In the MMP-9 gene expression graph, the authors observed an increase in MMP-9 gene expression using sgRNA1, and this increase was not seen in samples edited with sgRNA2. However, this same increase in protein expression was not seen; only a decrease in MMP-9 was seen in both edited samples compared to controls. Similar results were seen in another study by the studied group.^25^ The authors believe that this is a compensatory mechanism of the cell, in an attempt to increase the gene expression of MMP-9, already edited, to reestablish protein expression, compromised by the CRISPR technique. The authors did not find an absence of gene expression in all cases, because the cell can generate non-functional mRNA, but the sensitivity of the qPCR technique is capable of detecting it. Therefore, the authors suggest that this elevated MMP-9 gene expression is not from functional mRNA, based on the protein expression results.

MMP-9 is a 92-kDa gelatinase with proteolytic activity on collagen type IV.^26^ Its ECM activity interacts with many other targets, such as growth factors, cytokines, and chemokines, which in turn activate molecular pathways involved in cellular growth, invasion, inflammation, migration, and angiogenesis.^27^ MMP-9 activity is observed in many metastatic tumors, such as breast, esophageal, gastric, and prostate cancers.^16^^,^^17^ Several studies have demonstrated that MMP-9 is overexpressed in CaB patients compared with normal urothelial tissue^20^^,^^26^^,^^27^ and may be correlated to clinical prognostic factors such as high tumor pathological grade and size, stage, and progression.^19^^,^^28^^,^^29^ Moreover, several regulator proteins, growth factors, and cytokines, like IFN-γ, EGF, and TNF, exert their oncogenic effect by upregulating MMP-9,^30^, ^31^, ^32^, ^33^ suggesting its central role in cancer progression. In a previous study, non-metastatic bladder cancer cells transfected with IL-8 exhibited increased MMP-9 expression and invasive potential. Increased tumorigenicity and metastatic potential were detected when these cells were implanted into mice bladders, suggesting that upregulation of MMP-9 may induce invasive tumor growth and metastasis.^34^ In a similar experimental setting, the embryonic stem cell markers Oct-3/4 were also shown to increase MMP-9 expression, leading to the enhanced invasive and metastatic potential of bladder cancer cells.^35^ Another study demonstrated that MMP-9 activity reduction by abolishing the binding activity of essential transcription factors resulted in migration and invasion reduction in T24 cells.^36^ Similarly, downregulation of EMMPRIN (an extracellular MMP inducer, also known as CD147 represses MMP-9 expression, leading to decreased proliferation, migration, and invasion of bladder cancer cells.^37^ Therefore, MMP-9 is a potential diagnostic and prognostic biomarker, as well as a candidate for targeted therapeutic approaches.

Although the present findings are encouraging, some limitations should be acknowledged. The authors did not explore in detail the downstream molecular pathways related to MMP-9 suppression, nor did they conduct off-target analyses to exclude unintended CRISPR/Cas9 effects. Moreover, the absence of in vivo experiments and the variability observed in some assays restricts a broader translational interpretation of the results. Nonetheless, these limitations do not diminish the relevance of this study, which provides novel insights into the potential of CRISPR/Cas9-mediated MMP-9 silencing as a promising strategy to impair bladder cancer cell migration and invasion in vitro, paving the way for future mechanistic and preclinical investigations.

To our knowledge, the present study represents the first use of the CRISPR-Cas9 genome editing tool to perform MMP-9 knockout in bladder cancer cells and analyze its effects on bladder cancer cells. The present results were consistent with previous experimental studies aiming at MMP-9 inhibition or downregulation. For example, Dufour et al. developed a peptide that mimics the MMP-9 PEX domain blade IV, which reduced cell migration by blocking the requisite dimerization of MMP-9.^38^ More recently, the same group developed a low molecular weight compound that specifically binds to the PEX domain of MMP-9 and abolishes its homodimerization, blocking the CD44-AGFR-MAKP signaling pathway^38^ and thus interfering with MMP-9-mediated cancer cell proliferation, migration, and invasion.^39^ However, clinical trials focusing on MMP inhibitors showed no significant anti-tumor effects^40^, ^41^, ^42^ while presenting relevant adverse side effects.^42^ The initial therapeutic concept that general unspecific systemic inhibition of a broad spectrum of MMPs might significantly improve survival is too simplistic, as it does not consider the physiologic and non-ECM-degrading effects of MMP inhibition. Thus, theoretically, the high specificity and efficacy of CRISPR-Cas9 in blocking MMP-9 expression associated with tumor-specific transfection vectors may minimize the high volume of side effects observed with systemic MMP inhibitors. The studies cited, which minimized the expression of MMP-9, showed results but demonstrated limitations in inhibition, which were partial and lasted only for a short period of time. CRISPR-Cas9 is a recent and revolutionary technique, as it is possible to perform gene editing specifically in the region of interest, which in this study was MMP-9, and evaluate the evolution of the tumor for a longer period, without stimulating all the adjacent effects of current treatments. Multiple treatments are not necessary to evaluate the effects of inhibition. With few transfections, it is possible to perform permanent gene editing. We observed, with greater precision, that the absence of MMP-9, using the CRISPR-Cas9 technique, can minimize the migration and invasion capacity of edited cells, as well as increase the rate of total apoptosis, in relation to cells that normally express MMP-9. In addition, the possibility of intravesical therapy administration in BCa patients may also reduce the potential adverse effects.

## Conclusions

The present study demonstrated that inhibiting MMP-9 expression using the CRISPR-Cas9 genome editing technique is feasible and can successfully impair T24 bladder cancer cell migration and invasion capacities in vitro*.* It also corroborates the MMP-9 role in cancer cell migration and invasion processes. Thus, MMP-9 has an important effect on the progression of bladder cancer.

## Authors’ contributions

Gallucci, F.P: Formal analysis, investigation, methodology, writing-original draft. Camargo, J.A: Conceptualization; Data curation; formal analysis; methodology. Viana, NI: Conceptualization, methodology. Pimenta, R: Formal Analysis. Guimarães, V.R: Methodology. Candido, P: Methodology. Romão, P: Methodology. Leite, K.R.M: Project administration; supervision. Nahas, W.C: Supervision, visualization. Sabrina Reis: Conceptualization, supervision, visualization, writing-review & editing.

## Funding

This research did not receive any specific grant from funding agencies in the public, commercial, or not-for-profit sectors.

## Ethical approval and consent to participate

The manuscript does not require consent to participate as it does not have experiments with human and animal samples.

## Declaration of competing interest

The authors declare no conflicts of interest.

## Data Availability

The datasets used and/or analyzed during the current study are available to the corresponding author on reasonable request.
